# Not All Osteophytes Are Located on the Right Side of the Vertebrae in Diffuse Idiopathic Skeletal Hyperostosis: A Quantitative Analysis in Relation to the Position of Aorta

**DOI:** 10.1111/os.13869

**Published:** 2023-09-08

**Authors:** Haojie Chen, Qingshuang Zhou, Sinian Wang, Xiaojiang Pu, Haicheng Zhou, Bin Wang, Zezhang Zhu, Yong Qiu, Xu Sun

**Affiliations:** ^1^ Division of Spine Surgery, Department of Orthopaedic Surgery Nanjing Drum Tower Hospital, Affiliated Hospital of Nanjing University Medical School Nanjing China; ^2^ Division of Spine Surgery, Department of Orthopaedic Surgery Nanjing Drum Tower Hospital Clinical College of Jiangsu University Nanjing China

**Keywords:** Aorta, CT, Diffuse idiopathic skeletal hyperostosis, Morphology, Osteophyte, Position

## Abstract

**Objective:**

Diffuse idiopathic skeletal hyperostosis (DISH) is characterized by osteophytes in the anterior vertebrae, and the presence of aorta may have an impact on their formation. However, the anatomical positional relationship between the aorta and osteophytes in patients with DISH remains controversial. This study aimed to evaluate the position of osteophytes in relation to aorta in DISH, and the influence of aortic pulsation on the formation of osteophytes from the perspective of morphology.

**Methods:**

We conducted a retrospective review of 101 patients diagnosed with DISH and symptomatic lumbar spinal stenosis between June 2018 and December 2021. A total of 637 segments with heterotopic ossification in DISH were used for quantitative measurements on CT scans. The Cartesian coordinate system was built up on the axial CT scans to reflect the relative position between aorta and osteophytes. Osteophytes were divided into adjacent aorta group (AD group) and non‐adjacent aorta group (N‐AD group). In terms of the morphology, osteophytes in the AD group were further divided into convex, flat, and concave types. The relative position between aorta and osteophytes, and the aorta‐osteophyte distance and morphology of osteophytes were compared. Univariate analysis of variance was performed for multiple groups, and two independent‐samples *t*‐tests were used for two groups.

**Results:**

From T5 to L4, aorta gradually descended from left side to middle of vertebrae, and osteophytes gradually shifted from right side of vertebrae (T5‐T10) to bilateral sides (T11‐L4). Of 637 osteophytes in DISH, 60.1% (383/637) were in AD group, including convex type 0.6% (4/637), flat type 34.7% (221/637), and concave type 24.8% (158/637). The N‐AD group accounted for 39.9% (254/637). Flat osteophytes were concentrated in T5‐T12, while concave osteophytes in T11‐L4. Overall, the aorta‐osteophyte distance of concave type was significantly smaller than that of flat type.

**Conclusion:**

Osteophytes are not always located on the right side of vertebrae, but move with the position of the descending aorta. Furthermore, the morphology of osteophytes varies by vertebral segment in DISH, which is related to aorta descending anteriorly in the spine.

## Graphical abstract

DISH is characterized by anterior heterotopic ossification of spine, avoiding the aorta. Segments with osteophytes were grouped according to the morphology in the study, in order to investigate the positional relationship between the aorta and osteophytes and the influence of the aorta on the formation of the osteophytes in DISH.

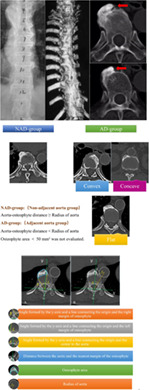

## Introduction

Diffuse idiopathic skeletal hyperostosis (DISH) is a systemic skeletal and muscular metabolic disorder of unknown etiology.^1^, ^2^ It is characterized by continuous osteophytes in four or more vertebral bodies, including ossification of ligaments and bone proliferation at entheses.^3^, ^4^, ^5^, ^6^ The prevalence of DISH was reported to be 3.9%–38.7% in selected populations.^7^, ^8^, ^9^, ^10^, ^11^ The thoracic spine is the most commonly affected site of DISH in the early stages, followed by the lumbar and cervical spine.^4^, ^12^ Bone deposition in the spine of patients with DISH is regularly asymptomatic, but it can lead to gradually stiffening of the spine, airway obstruction, dysphagia, and symptoms of nerve compression.^13^

The typical osteophyte changes associated with DISH occur most frequently in the anterolateral part of the thoracic spine. Osteophytes were reported to be located contralaterally to the descending thoracic aorta.^14^, ^15^ It has been hypothesized by some previous studies that the mechanical pressure created by persistent aortic pulsations could inhibit soft tissue mineralization^6^ and affect the distribution of osteophytes in DISH.^3^, ^14^, ^15^ Kuperus *et al*.^16^ reported that osteophytes were evidently located more on the right side of the thoracic spine for the segments T3 to T12, while the position of osteophytes was on the left side of the spine in DISH patients with splanchnic inversion.^17^, ^18^ Whereas, Castells Navarro and Buckberry^4^ addressed a different finding that the newly formed osteophytes were distributed on both sides from segments T10 to L5 in gross specimens of DISH. These confounding results may be related to the various positions of the migratory aorta at each vertebral level.^19^ Therefore, it is necessary to further analyze the positional relationship between the aorta and osteophytes in different vertebral segments of DISH.

In DISH patients, the morphology of osteophytes could potentially indicate the influence of aortic pulsation on their formation, in addition to their positioning relative to the vertebra. Mori *et al*.^20^ have reported that aortic pulsation can affect the morphology of osteophytes on axial CT imagines. The osteophyte in DISH was likely to continue to form and remodel.^16^ If aortic pulsation can inhibit the formation of DISH osteophytes, it may also affect the size and morphology of osteophytes. Limited research has been conducted on the correlation between the positioning of the aorta and osteophytes, as well as the morphology of osteophytes, in patients diagnosed with DISH.

Therefore, we performed the current study with the aim to investigate the spatial positions of osteophytes in relation to the aorta in DISH, and to evaluate the effect of aortic pulsation on the formation of osteophytes in DISH from the perspective of morphology on the axial CT images.

## Materials and Methods

### Study Sample

This study was approved by the Ethics Committee (Ethical number: 2021‐398‐01). We reviewed a consecutive series of patients with DISH who had undergone instrumented fusion and decompression for lumbar spinal stenosis between June 2018 and December 2021 in our center.

Inclusion criteria for this study were as follows: (i) patients aged between 50 ~ 80 years who were diagnosed with DISH; and (ii) had complete thoracic and lumbar CT reconstruction scans before surgery. Exclusion criteria were as follows: (i) previous history of spinal surgery; (ii) had spinal fractures, scoliosis>10°, ankylosing spondylitis (AS), spinal tumors, and spinal infection. The existence of DISH was confirmed in multiple planes of CT, including replanning of axial and sagittal sections, and three‐dimensional reconstruction.

### Diagnostic Criteria of DISH

The diagnostic criteria of DISH were established by Resnick and Niwayama.^14^ These criteria required the involvement of at least four adjacent vertebrae in the spine, preservation of intervertebral disc space, and the absence of significant degeneration or fusion of the apophyseal and sacroiliac joints. To optimize the assessment of DISH, we selected the sagittal plane of the spine through both X‐ray and CT imaging. Three experienced spinal surgeons (X.S., B.W. and Y.Q.) were responsible for diagnosing patients with DISH. When there was uncertainty about DISH and AS, some additional tests needed to be performed, including erythrocyte sedimentation rate, C‐reactive protein, human leukocyte antigen‐B27, and sacroiliac joint CT.

### CT Scan and Reconstruction

All CT scans were performed with a spiral CT scanner (Lightspeed 16, GE Medical Systems, Milwaukee, WI), according to the following protocol: 120 kV; tube current 100–350 mA; gantry rotation 0.8 s; feed 27.5 mm/rotation; and slice thickness and detector collimation of 1.25 and 5 mm, respectively. Axial and sagittal sections and three‐dimensional images were obtained.

### Radiographic Assessment

**FIGURE 1:**
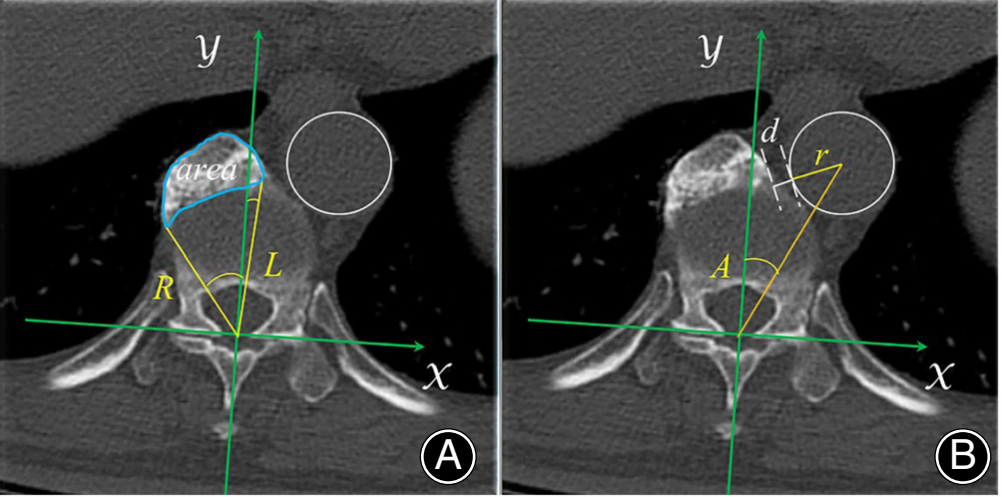
The parameters measured on an axial CT scan. (A) area: the area of osteophyte at the selected scan. R & L: angle separately formed by the y‐axis and a line connecting the origin and the right and left margin of osteophyte. (B) A: angle formed by the y‐axis and a line connecting the origin and the center to the aorta. d: the distance between the aorta and the nearest margin of the osteophyte. r, the radius of the aorta.

Since the anterolateral osteophyte area of DISH was the largest in the intervertebral space as reported by Verlaan *et al*.,^3^ the axial images were selected for the measurement in the intervertebral disc level, which could view the bases of the inferior facet. To quantify the relative position of the aorta and paravertebral osteophyte, a Cartesian coordinate system was built up on the axial CT scans of the vertebral bodies referring to the methods of Feng *et al*.^19^ (Figure 1). The origin was set at the middle of the base of two inferior facets. A line joining the middle points of both bases of the inferior facets was defined as the x‐axis. The y‐axis, perpendicular to the x‐axis, was drawn ventrally from the origin. (Figure 1).

To assess the relationship between the position of the aorta and osteophytes, the following parameters were measured at each level from T4 to L4, taking into account both the presence of aorta running and continuous ossification in front of the vertebrae (Figure 1): (i) angle R & L, separately subtended by the y‐axis and a line connecting the right and left margin of osteophyte; and (ii) angle A: formed by the y‐axis and a line connecting the center of the aorta. In addition, the aorta‐osteophyte nearest distance (d), radius of the aorta (r) and osteophyte area were measured simultaneously. Angle between the right margin of the osteophytes and the y‐axis was defined as negative.

### Morphological Grouping of Osteophytes

**FIGURE 2:**
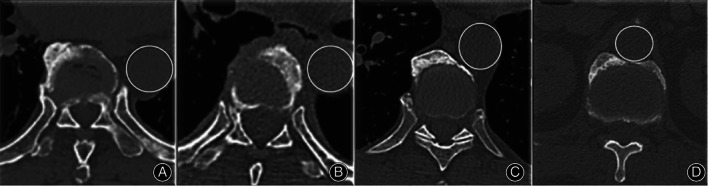
Graphical illustration of osteophyte grouping. (A) Segments with aorta‐osteophyte distance ≥ aortic radius (NAD group). (B–D) Segments with aorta‐osteophyte distance < aortic radius (AD group). (B) Convex; (C) Flat; (D) Concave.

According to the study of Mori *et al*.,^20^ segments with aorta‐osteophyte distance ≥ aortic radius were included in the non‐adjacent aorta group (NAD group). Segments with aorta‐osteophyte distance < aortic radius were assigned to the adjacent aorta group (AD group). We investigated the shape of osteophyte adjacent to the aorta and divided osteophytes into three types in AD group: convex, flat, and concave^20^ (Figure 2).

All radiographic assessments and morphological grouping of osteophytes were reviewed by two observers independently and disputes were resolved by a third observer. A total of 40 CT scans were randomly selected by another independent investigator for repeated morphological grouping of osteophytes, which were done at least 1 month after the initial assessment.

### Intra and Interrater Reliability Study

The Kappa consistency test, also known as Cohen's Kappa, was analyzed for intrarater reliability. Fleiss’ kappa coefficient was analyzed for interrater reliability. The first morphological grouping of osteophytes in all observers was analyzed for interrater reliability. Kappa values were analyzed as follows: less than 0 for no agreement, 0–0.20 for slight, 0.21–0.40 for fair, 0.41–0.60 for moderate, 0.61–0.80 for substantial, and 0.81–1.0 for near‐perfect, respectively.

### Statistical Analysis

All radiographic parameters were measured three times by Surgimap spine 2.3.2 (Nemaris Company, New York, USA) and the average value was used for further analyses. SPSS Statistics (v26.0; IBM Corp., Armonk, NY, USA) was used for statistical analysis. The Shapiro–Wilk method was used to estimate the normal distribution of data. All continuous variables (angle L, angle R, angle A, osteophyte area and aorta‐osteophyte distance) were in accordance with normal distribution in each spine level and presented as means with standard deviation (SD). Univariate analysis of variance (ANOVA) was used for multiple groups, and two independent‐samples t tests for two groups.

First, angle A, angle R and angle L were tested in each segments by the univariate ANOVA. Second, Kappa consistency test of intrarater and interrater was evaluated for morphological grouping of osteophytes. Third, the comparisons of both osteophyte area and aorta‐osteophyte distance between flat and concave osteophytes were compared by paired‐*t* test regardless of segments. Last, the comparison of aorta‐osteophyte distance between flat and concave osteophytes was performed by paired‐*t* test at each segment. *p* value <0.05 indicated statistical significance.

## Results

As shown in Table 1, a total of 101 patients (65 males and 36 females), whose average age was 66 years (range, 52–79 years), with 637 spinal ossified segments were included in the study.

**TABLE 1:** Characteristics of patient population

Number of patients	101
Sex (male/female)	65/36
Mean age (years)	66.0 ± 10.3
BMI (kg/m^2^)	26.8 ± 3.3
Mean number of ossified segments	7.5 ± 2.2

### The Spatial Positional Relationship between Aorta and Osteophytes

**FIGURE 3:**
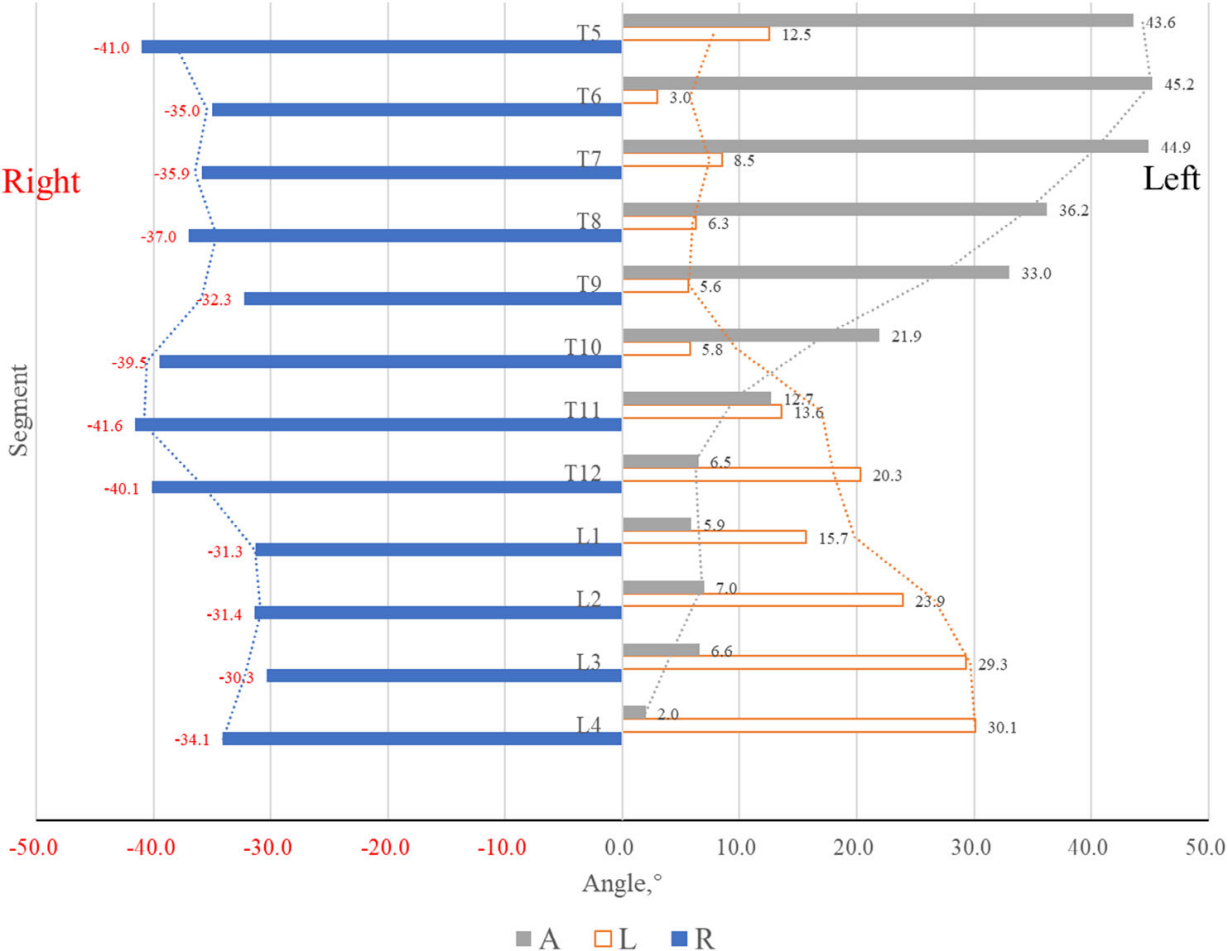
The spatial position of the aorta and osteophytes.

The positioning of the aorta in relation to the osteophytes in various segments of the spine was illustrated in Figure 3. The continuous variables (angle L, angle R and angle A) were in accordance with normal distribution in each spine level. From T5 to L4, angle A decreased gradually from 43.6° to 2.0°, indicating the aorta moved from the left to the middle of the vertebra. Angle L increased from 12.5° to 30.1°, while angle R had no obvious trend of change, suggesting the left margin of the osteophyte developed to the left, but the right margin did not change. From T5 to T10, angle A was significantly larger than both angle R and angle L (*p*<0.05). Whereas, from T11 to L4, angle A was significantly larger than angle R, but significantly smaller than angle L (*p*<0.05), indicating that the relative spatial position of the aorta and osteophytes varied with the migration of the aorta at different segments. In general, the aorta was positioned more anterolaterally at the T4‐T10 levels, while more anteromedially at the T11‐L4 levels. Correspondingly, the location of osteophytes also shifted from the right side of the aorta at T4‐T10 levels to the both sides at T11‐L4 levels.

### Reliability of Osteophyte Morphological Grouping

The intrarater reliability analysis for morphological grouping of osteophytes demonstrated κ values of 0.85 for all osteophytes. The interrater reliability analysis demonstrated κ values of 0.82 for all osteophytes.

### Distribution of Osteophyte Morphological Grouping

The distribution of each osteophyte group is highlighted in Table 2. Overall, the NAD group accounted for 39.9% (254 of 637), and in the AD group, flat osteophytes accounted for 34.7% (221 of 637), followed by concave 24.8% (158 of 637) and convex 0.6% (4 of 637). In terms of segmental distribution, osteophytes in the NAD group mainly located in T5 to T10. In the AD group, convex osteophytes were found in only four cases, flat osteophytes were commonly detected in T5 to T12, and concave osteophytes were frequently observed in T11 to L4.

**TABLE 2:** The segmental distribution of osteophytes in each group

Segment	No	NAD group n (%)	AD group n (%)		
			Convex	Flat	Concave
T5	33	13 (39.4)	0 (0)	20 (60.6)	0 (0)
T6	50	36 (72.0)	0 (0)	14 (28.0)	0 (0)
T7	69	47 (68.1)	2 (2.9)	18 (26.1)	2 (2.9)
T8	74	54 (73.0)	0 (0)	16 (21.6)	4 (5.4)
T9	79	47 (59.5)	0 (0)	29 (36.7)	3 (3.8)
T10	77	30 (39.0)	0 (0)	45 (58.4)	2 (2.6)
T11	74	13 (17.6)	2 (2.7)	29 (39.2)	30 (40.5)
T12	57	5 (8.8)	0 (0)	23 (40.3)	29 (50.9)
L1	52	7 (13.5)	0 (0)	9 (17.3)	36 (69.2)
L2	36	0 (0)	0 (0)	11 (30.6)	25 (69.4)
L3	23	2 (8.7)	0 (0)	5 (21.7)	16 (69.6)
L4	13	0 (0)	0 (0)	3 (23.1)	10 (76.9)
All	637	254 (39.9)	4 (0.6)	221 (34.7)	158 (24.8)

### Comparison of Radiographic Parameters in Different Osteophyte Groups

**FIGURE 4:**
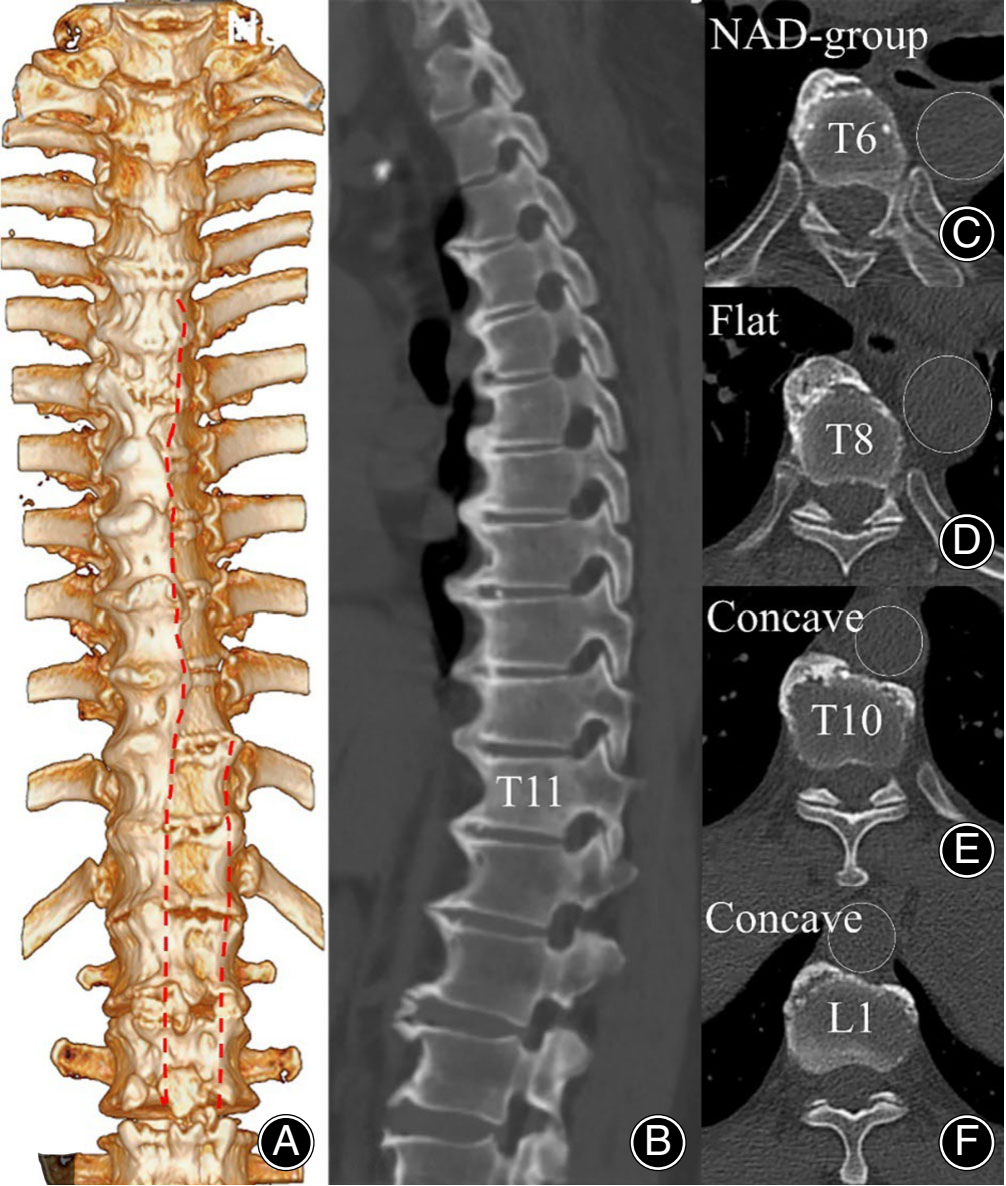
The CT 3‐D reconstructed anterior view, sagittal plane, and axial images of a thoracolumbar spine in a 71‐year‐old male patient with DISH. (A) The track of the aorta on the spinal osteophyte (the red dotted line); (B) Sagittal images illustrating osteophytes of varied morphology on CT; (C) Osteophyte of NAD group; (D) Flat osteophyte; (E and F) Concave osteophyte.

**FIGURE 5:**
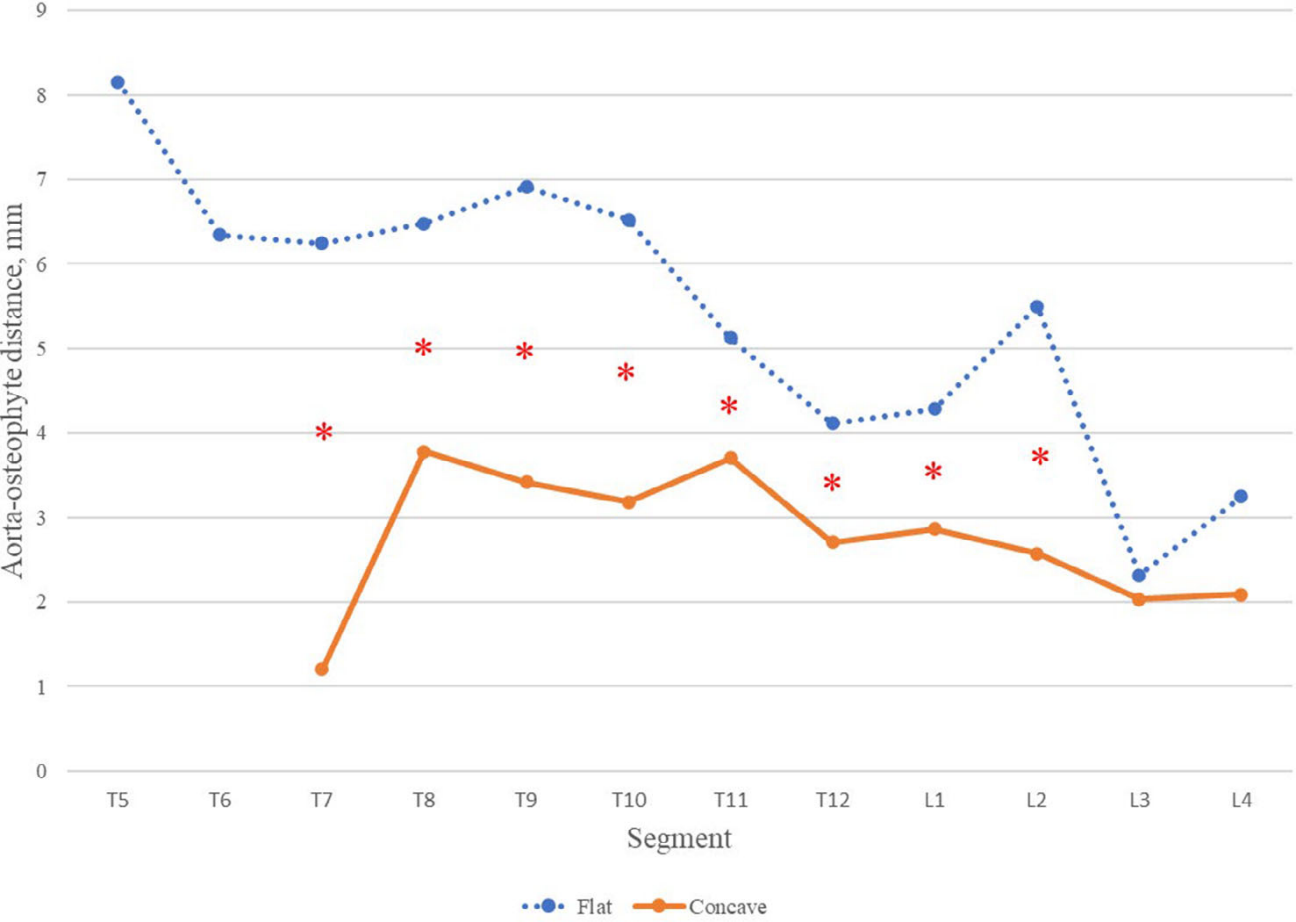
Comparison of aorto‐osteophyte distance between flat and concave osteophyte in different segments. *p<0.05.

The radiographic parameters of different osteophyte groups are shown in Table 3. The osteophyte area and aorta‐osteophyte distance in the NAD group were significantly smaller than both flat and concave osteophytes of the AD group. The aorta‐osteophyte distance in flat osteophytes was significantly larger than that in concave osteophytes (5.2 ± 2.1 vs.3.2 ± 2.1mm, *p*<0.01), while there was no significant difference in osteophyte area between flat and concave osteophytes (252.8 ± 123.1 vs.284.6 ± 129.6mm^2^, *p* = 0.174). As shown in Figures 4 and 5, the aorta‐osteophyte distance of flat osteophytes is significantly larger than that of concave in most segments, including T7, T8, T9, T11, T12, L1, and L2. It means that the aorta was more closer to the concave than flat osteophytes (Figure 5).

**TABLE 3:** Parameter information of each group

	NAD group, mean±SD	AD group, mean±SD			NAD group vs. flat	NAD group vs. concave	Flat vs. concave
		Convex	Flat	Concave			
Segments, n	254	4	221	158	‐	‐	‐
Osteophyte area (mm^2^)	172.1 ± 92.6	135.3 ± 57.2	252.8 ± 123.1	284.6 ± 129.6	<0.001	<0.001	0.282
Aorta‐osteophyte distance (mm)	21.5 ± 6.7	6.4 ± 2.2	5.2 ± 2.1	3.2 ± 2.1	<0.001	<0.001	<0.001

## Discussion

In this study, we quantitatively investigated the relative spatial position of segmental osteophytes and aorta in patients with DISH. Our results revealed that the locations of osteophytes in DISH patients did not always located on the right side of the vertebral body, but changed with the position of the descending aorta. Additionally, the morphology of osteophytes in DISH patients varied by vertebral segments, which was related to the aorta descending anteriorly in the spine.

### The Spatial Positional Location between Aorta and Osteophytes

The anatomical positioning of the aorta in relation to the anterior ossification tissue in each segment of the spine may vary among patients with DISH. Castells Navarro and Buckberry^4^ have found in archeological gross specimens that osteophytes in patients with DISH were inclined to the right from T5 to T10, and osteophytes were usually divided into two segments and distributed on both sides of the vertebra from T10 to L5. Their findings were similar to our study from a radiographic perspective. In our study, from T5 to L4, the aorta gradually descended from the left side to the middle of the vertebrae, the osteophytes gradually shifted from the right side alone of the vertebra (T5‐T10) to both right and left sides of the vertebra (T11‐L4), and the left margin of the osteophyte shows progression towards the left, while the right margin remains unchanged (Figure 3). In the 3‐D CT reconstruction of the spine in DISH patients (Figure 4), the aorta is clearly traced in the osteophytes in front of the spine. This vividly figured out that the relative spatial position of the aorta and osteophytes in DISH patients varied in different vertebral levels.

Previous studies have focused on thoracic vertebra osteophytes occurring on the right side of the spine and opposite to the aorta,^21^, ^22^ and then speculated that pulsating aorta inhibited osteophytes. Yet, our study showed that osteophytes in DISH were not always located on the right side at all vertebral segments. It may not be sufficient to explain the effects of the aorta on osteophytes formation only based on the positional relationship between the aorta and osteophytes. Mori *et al*.^20^ reported that aortic pulsation could affect the morphology of osteophytes, which indicated the aortic pulsation might play an important role in the formation of osteophytes in DISH. Therefore, the effect of aorta on the morphology of osteophytes should not be ignored.

### Association between Osteophytes Morphology and Relative Position of Aorta and Spine

The morphological distribution of osteophytes varied with segment in patients with DISH. In this study, flat and concave osteophytes were mainly found in the AD group (Table 2). Convex osteophytes occurred in only four cases. The reason why convex osteophytes were close to the aorta was not clear. Mori *et al*.^20^ thought that it was caused by insufficient pressure of aortic pulsation. Flat and concave osteophytes are common in the lower thoracic and lumbar vertebra, which may be related to the proximity of the aorta to the surface of the vertebra. DISH osteophytes often originate from the lower thoracic vertebra and then spread to the upper thoracic and lumbar vertebrae.^23^ The aorta is commonly observed to be positioned to the left of the thoracic vertebrae, potentially resulting in the displacement of osteophytes towards the right side. This unique phenomenon contributes to the concentration of DISH osteophytes along the right margin of the thoracic vertebrae.

Three forms of osteophytes have been reported in patients with DISH, and osteophytes adjacent to the aorta were mostly flat and concave, suggesting that mechanical stress induced by aortic pulsation may play a crucial role in the development of osteophytes in DISH.^20^ On the basis of previous studies, the aorta‐osteophyte distance in different osteophyte morphologies was analyzed in this study, and we found that the aorta‐osteophyte distance of concave osteophytes was smaller than flat osteophytes, indicating that the aorta may impact on the morphology of osteophytes in DISH. However, the area of the concave osteophyte was not significantly different from that of the flat type, even the average area of the concave osteophytes was larger than that of the flat type (Table 3). This suggests that the aorta may only influence the morphology of osteophytes and is not necessarily involved in the formation of osteophytes in DISH.

### Findings and Clinical Relevance of the Study

Our study mainly focused on the analysis of the difference between flat and concave osteophytes in the AD group. It was found that in most segments, the aorta‐osteophyte distance of flat type was significantly larger than concave type (Figure 5), which further described that aortic pulsation could impact on the formation of osteophytes, and influence the morphology of osteophytes in DISH. Although the current work does not provide direct histological evidence, the hypothesis suggesting that the aorta influences new bone formation in the anterior spinal column in DISH remains highly compelling. Due to the difficulty in obtaining anterior vertebral osteophytes during surgery, our study was the first to provide substantial evidence for the influence of aorta on the formation of osteophytes in DISH patients from an imaging perspective.

This study may provide some new references for the diagnosis of DISH. There are osteophytes in four consecutive segments of the vertebrae in DISH patients, and the osteophytes adjacent to the aorta are flat or concave on the axial CT images. The establishment of the optimal pulsating stress condition for simulating aortic pulsation and its transmission system may slow the formation or change the shape of osteophytes in DISH. In the future, the effects of aortic pulsation on osteophyte formation from the perspective of molecular biology still need to be further explored.

### Limitations

There are several limitations to this study. First, CT was taken in the supine position, and the position of aorta was different from that in standing position. So we were unable to analyze the effect of hydrostatic pressure at different segments. Second, this study lacked DISH patients who received conservative treatment. Third, Cervical osteophytes were not analyzed in our study. There was no aorta in the cervical vertebra region. Further investigation is needed to understand the potential influence of the cervical vertebral arteries on osteophyte formation.

## Conclusion

Osteophytes in DISH patients are not always located on the right side of the vertebrae, but move with the position of the descending aorta. Furthermore, the morphology of osteophytes varies by vertebral segment in DISH patients, which is related to the aorta descending anteriorly in the spine. When the aorta moves closer to the osteophytes, the osteophytes exhibit a concave shape. Conversely, when the aorta moves away from the osteophytes, the osteophytes appear flat.

## Author Contributions

H.C., Q.Z. and S.W. reviewed radiographs. H.C. performed statistical analysis and drafted the manuscript. X.P., H.Z., B.W. and Z.Z. gave administrative and intellectual support. X.S. and Y.Q. conceived the study, finalized the manuscript and is responsible. All authors read and approved the final manuscript.

## Ethics Statement

All procedures involving participants and data collection were approved by ethics committee of Nanjing Drum Tower Hospital prior commencement of the study. Informed consent was obtained from all participants prior to their inclusion in the study. We would like to thank all participating patients.
